# Highly Divergent Genetic Variants of Soricid-Borne Altai Virus (*Hantaviridae*) in Eurasia Suggest Ancient Host-Switching Events

**DOI:** 10.3390/v11090857

**Published:** 2019-09-14

**Authors:** Hae Ji Kang, Se Hun Gu, Liudmila N. Yashina, Joseph A. Cook, Richard Yanagihara

**Affiliations:** 1 John A. Burns School of Medicine, University of Hawaii at Manoa, Honolulu, HI 96813, USA; hyeyun.kang@gmail.com (H.J.K.); sehun.gu@gmail.com (S.H.G.); 2 State Research Center of Virology and Biotechnology, “Vector”, Koltsovo 630559, Russia; yashina@vector.nsc.ru; 3 Museum of Southwestern Biology, University of New Mexico, Albuquerque, NM 87131, USA; cookjose@unm.edu

**Keywords:** *Hantaviridae*, hantavirus, shrew, viral evolution

## Abstract

With the recent discovery of genetically distinct hantaviruses (family *Hantaviridae*) in shrews (order Eulipotyphla, family *Soricidae*), the once-conventional view that rodents (order Rodentia) served as the primordial reservoir hosts now appears improbable. The newly identified soricid-borne hantaviruses generally demonstrate well-resolved lineages organized according to host taxa and geographic origin. However, beginning in 2007, we detected sequences that did not conform to the prototypic hantaviruses associated with their soricid host species and/or geographic locations. That is, Eurasian common shrews (*Sorex araneus*), captured in Hungary and Russia, were found to harbor hantaviruses belonging to two separate and highly divergent lineages. We have since accumulated additional examples of these highly distinctive hantavirus sequences in the Laxmann’s shrew (*Sorex caecutiens*), flat-skulled shrew (*Sorex roboratus*) and Eurasian least shrew (*Sorex minutissimus*), captured at the same time and in the same location in the Sakha Republic in Far Eastern Russia. Pair-wise alignment and phylogenetic analysis of partial and full-length S-, M- and/or L-segment sequences indicate that a distinct hantavirus species related to Altai virus (ALTV), first reported in a Eurasian common shrew from Western Siberia, was being maintained in these closely related syntopic soricine shrew species. These findings suggest that genetic variants of ALTV might have resulted from ancient host-switching events with subsequent diversification within the Soricini tribe in Eurasia.

## 1. Introduction

Thottapalayam virus (TPMV), a previously unclassified virus isolated from an Asian house shrew (*Suncus murinus*), captured in southern India in 1964 [1], predated the discovery of Hantaan virus (HTNV), the prototype virus of hemorrhagic fever with renal syndrome (HFRS), in the striped field mouse (*Apodemus agrarius*) in Korea by more than a decade [2]. However, this observation went largely unnoticed and the subsequent detection of HFRS antigens in tissues of the Eurasian common shrew (*Sorex araneus*) and Eurasian water shrew (*Neomys fodiens*) in European Russia and the former Yugoslavia [3,4,5] similarly failed to incite systematic exploration into the role of shrews in the evolutionary origins of hantaviruses. 

Guided by these decades-old reports and spurred by the fortuitous availability of tissues from Ussuri white-toothed shrews (*Crocidura lasiura*), captured coincidentally as part of a HTNV surveillance program along the Imjin River near the demilitarized zone in South Korea, a genetically distinct hantavirus sharing a common ancestry with TPMV, named Imjin virus (MJNV), was isolated in 2008 [6,7]. Thereafter, armed with the whole genome of the newfound MJNV and empowered by the generosity of museum curators and field mammalogists, who provided access to their collections of shrew tissues, we launched an opportunistic search for hantavirus RNA using reverse transcription polymerase chain reaction (RT-PCR) [8,9]. In analyzing more than 1500 frozen, RNAlater^®^-preserved and ethanol-fixed archival tissues from more than 50 shrew species (order Eulipotyphla, family Soricidae, subfamilies Soricinae, Crocidurinae and Myosoricinae) captured in Europe, Asia, Africa and North America during the past three decades (1980–2013), we have discovered multiple novel hantaviruses, which are more genetically diverse than those harbored by rodents [10,11,12,13,14,15,16,17,18,19,20,21].

Among the shrew species in which HFRS antigens were originally detected more than 30 years ago, confirmation by RT-PCR detection of hantavirus RNA was first achieved in the Eurasian common shrew [12]. The hantavirus, named Seewis virus (SWSV), after the capture site in the Swiss canton of Graubünden, has since been detected in Eurasian common shrews across much of its vast geographical range, in Austria [22], Czech Republic [22], Finland [23,24,25], Germany [22], Hungary [23], Poland [17,26], Russia [27], Slovakia [22] and Slovenia [28,29]. Possibly as evidence of spill-over events, SWSV has also been detected in the Eurasian pygmy shrew (*Sorex minutus*) [17,22,26], Mediterranean water shrew (*Neomys anomalus*) ([26], GenBank EU418604), Siberian large-toothed shrew (*Sorex daphaenodon*) [27] and tundra shrew (*Sorex tundrensis*) [27].

Phylogenetic analysis of SWSV and other newly identified soricid-borne hantaviruses show well-resolved lineages organized largely by host taxa and geographic origin [8,9]. However, beginning in 2007, we detected hantavirus sequences that did not conform to their reservoir soricid host species and/or geographic location. That is, within populations of Eurasian common shrews, captured at the same time and in the same locations in Hungary and Russia, we found co-circulation of a highly divergent hantavirus lineage. Pair-wise alignment and comparison of a 300-nucleotide region of the L segment indicated that a distinct hantavirus species was being maintained. Not knowing what these sequences signified, we initially chose not to report them, except to deposit one of these sequences, designated Altai virus (ALTV) strains Telet-Sa302 (GenBank EU424341), amplified from tissues of a Eurasian common shrew, captured near Teletskoye Lake in the Altai Republic in August 2007 [27].

Finding many more examples of these highly distinctive hantavirus sequences from syntopic *Sorex* species in Far Eastern Russia during the ensuing years forms the basis of this report. Also, in including expanded sequences of ALTV and previously unreported ALTV-like hantavirus sequences from two Eurasian common shrews from Hungary and one Eurasian pygmy shrew from Poland, as well as the recently reported full-length genome of Lena River virus (LENV) strain Khekhtsir-Sc67 from a Laxmann’s shrew (*Sorex caecutiens*) in Far Eastern Russia [30], we demonstrate that this presumptive soricid-borne hantavirus species might represent an ancestral lineage that subsequently diversified within the Soricini tribe in Eurasia. Because the ramifications of this conjecture are far reaching, more intensive research is urgently needed, including the isolation and characterization of ALTV and ALTV-like hantaviruses, to establish their contribution to the evolutionary history of hantaviruses and their proper placement in the new taxonomic classification of the family *Hantaviridae* [31].

## 2. Materials and Methods

### 2.1. Trapping and Sample Collection

**Figure 1:**
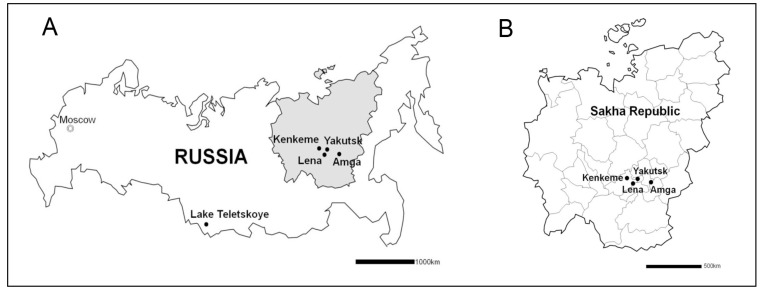
Maps showing (**A**) the location of the Sakha Republic in Far Eastern Russia, and (**B**) the small mammal collection sites in the Sakha Republic, where hantavirus-infected *Sorex* shrews were captured in July and August 2006.

Shrew specimens were collected, as part of the Beringian Coevolution Project [32], along the Amga River, 10 km NE Sulgachi (61.58046/133.14386), 7 km N Sulgachi (61.59218/132.93862) and 8 km ENE Mikhaylovka (61.24610/132.71483); Kenkeme River, 40 km W Yakutsk (62.07003/128.93831); and Lena River, 2 km NW Tochtur (61.75421/129.52548), near Yakutsk, the capital of the Sakha Republic in Siberian Russia, during July and August 2006. These samples are indicated in bold type in Table 1, and collection sites are shown in Figure 1. Table 1 also summarizes the prevalence of hantavirus RNA previously reported for soricine shrews from selected regions in Finland [23], Hungary [23], Poland [26] and Russia [27]. Field procedures and protocols, including trapping, euthanasia and tissue processing, were performed, following the animal care and use guidelines of the American Society of Mammalogists [33] and were approved by the Institutional Animal Care and Use Committee, of the University of New Mexico (protocol 06UNM026). Standard museum vouchers were prepared, with samples of lung frozen in liquid nitrogen for transport to the Museum of Southwestern Biology, where tissues were archived at −80 °C and associated databases are maintained to foster pathobiology research [34].

### 2.2. RNA Extraction and RT-PCR Analysis

Total RNA was extracted from lung tissues, using the PureLink Micro-to-Midi total RNA purification kit (Invitrogen, San Diego, CA, USA), then reverse transcribed, using the SuperScript III First-Strand Synthesis System (Invitrogen) with random hexamers and universal oligonucleotide primer (OSM55, 5′-TAGTAGTAGACTCC-3′), designed from the conserved 3′ end of the S and L segments of hantaviruses [15,16,20,26]. Oligonucleotide primers used to amplify the S-, M- and L-genomic segments are provided in Table S1. For the amplification of hantavirus genes, a two-step PCR was performed in 20-μL reaction mixtures, containing 250 μM dNTP, 2 mM MgCl_2_, 1 U of AmpliTaq polymerase (Roche, Basel, Switzerland) and 0.25 μM of each oligonucleotide primer [14,20,23,27]. Initial denaturation at 94 °C for 5 min was followed by two cycles each of denaturation at 94 °C for 40 s, two-degree step-down annealing from 48 to 38 °C for 40 s, and elongation at 72 °C for 1 min, then 32 cycles of denaturation at 94 °C for 40 s, annealing at 42 °C for 40 s, and elongation at 72 °C for 1 min, in a GeneAmp PCR 9700 thermal cycler (Perkin-Elmer, Waltham, MA, USA). Amplicons were separated by electrophoresis on 1.5% agarose gels and purified using the QIAQuick Gel Extraction Kit (Qiagen, Hilden, Germany). DNA was sequenced directly using an ABI Prism 377XL Genetic Analyzer (Applied Biosystems, Foster City, CA, USA).

### 2.3. Genetic Analysis

Pair-wise alignment and comparison of partial and full-length S- and L- and partial M-segment sequences of newfound hantaviruses from soricine shrews with representative rodent-, shrew-, mole- and bat-borne hantaviruses were performed, using the ClustalW method (TranslatorX server and BioEdit 7.0.5) [35,36,37]. In addition, we reanalyzed the previously reported and unreported hantavirus sequences from archival tissues of Eurasian common shrews captured in Györ-Sopron-Moson (SWSV Sa105/MSB95462, SWSV Sa106/MSB95461, SWSV Sa202/MSB95464, SWSV Sa211/MSB95480) and Zala (SWSV Sa244/MSB94609) in Hungary [23] and the Altai Republic (SWSV Telet-Sa300, SWSV Telet-Sa301, SWSV Telet-Sa313, SWSV Telet-Sa321, SWSV Telet-Sa500) in Russia [27], and included the 278- and 4,997-nucleotide S- and L-segment sequences, respectively, of ALTV Telet-Sa302, previously amplified, but not reported, from lung tissue of a Eurasian common shrew, as well as the ALTV-like hantavirus partial S-segment sequences amplified from lung tissue of a Laxmann’s shrew captured in Krasnoyarsk Krai in August 2008 (Parnaya-Sc1217), the ALTV-like hantavirus full-length genome from Laxmann’s shrew in Khabarovsk Krai in February 2008 (Khekhtsir-Sc67) and the ALTV-like hantavirus partial L-segment sequence from a Eurasian pygmy shrew captured in Chmiel, Poland, in September 2010 (Smin1108).

### 2.4. Recombination Analysis

Nucleotide sequences of the coding regions, including the full-length L segment, were analyzed using multiple recombination-detection methods [38,39], including GENECONV, Bootscan, Chimaera, 3SEQ, RDP, SiScan, MaxChi and HyPhy Single Recombinant Breakpoint, within the RDP4 Beta 4.36 software (http://web.cbio.uct.ac.za/~darren/rdp.html).

### 2.5. Phylogenetic Analysis

The maximum likelihood and Bayesian methods, implemented in RAxML Blackbox webserver [40] and MrBayes 3.1 [41], under the best-fit general time reversible model of nucleotide evolution with gamma-distributed rate heterogeneity and invariable sites (GTR+I+Γ) [42] and jModelTest version 0.1 [43], were used to generate phylogenetic trees. Two replicate Bayesian Metropolis–Hastings Markov Chain Monte Carlo (MCMC) runs, each consisting of six chains of 10 million generations sampled every 100 generations with a burn-in of 25,000 (25%), resulted in 150,000 trees overall. The S and L segments were treated separately in phylogenetic analyses. Topologies were evaluated by bootstrap analysis of 1000 iterations, and posterior node probabilities were based on 2 million generations and estimated sample sizes over 100 (implemented in MrBayes).

### 2.6. Mitochondrial DNA (mtDNA) Host Phylogeny

The taxonomic identity of the hantavirus-infected shrews was verified in genomic DNA extracted from tissues using the QIAamp DNA Mini Kit (Qiagen) and their phylogenetic relationships were studied by analysis of the complete 1140-nucleotide cytochrome b gene, amplified by PCR using well-tested primers (forward: 5′-CGAAGCTTGATATGAAAAACCAT CGTTG-3′; and reverse: 5′-CTGGTTTACAAGACCAGAGTAAT-3′) [44]. Host phylogenies based on mtDNA cytochrome *b* sequences, along with published sequences for shrews and moles for this gene region, were generated, using the maximum-likelihood and Bayesian methods described previously [45,46]. The tree was based on 3,000,000 MCMC generations, sampled every 100 generation and burn-in after 10,000 trees.

### 2.7. Virus Isolation

Using the previously described methods [6,47], 1% and 10% (*w*/*v*) homogenates of lung tissues from *Sorex* shrews, confirmed as infected with hantavirus by RT-PCR and sequencing, were inoculated onto subconfluent monolayers of Vero E6 cells (ATCC C1008 CRL-1586, American Type Culture Collection, Manassas, VA), grown in 25-cm^2^ flasks and maintained with Dulbecco’s minimum essential medium containing 5% fetal bovine serum. Cells were subcultured at two- to four-week intervals, at which time aliquots of cells were examined for hantavirus RNA by RT-PCR. Blind passages of cells were conducted for more than 100 days.

## 3. Results

### 3.1. RT-PCR Detection of Hantavirus RNA

Hantavirus RNA was detected by RT-PCR and confirmed by DNA sequencing in lung tissues collected from 15 of 49 Laxmann’s shrews, four of 12 flat-skulled shrews, one of five Eurasian least shrews, and in none of five tundra shrews and four Siberian large-toothed shrews (Table 1). The majority of captured shrews were male (35 of 49 Laxman’s shrews, eight of 12 flat-skulled shrews, and five of five Eurasian least shrews), and 17 of the 20 hantaviruses were found in male shrews, but the difference was not statistically significant (Fisher exact test value, 0.2287; *p* > 0.05). Of the 20 *Sorex* shrew-borne hantaviruses from the Sakha Republic, 14 resembled ALTV, instead of the expected host-specific hantavirus (SWSV and KKMV) (Table 2).

### 3.2. Genetic Analysis

Table 2 summarizes the S-, M- and L-segment hantavirus sequences obtained for each of the 20 hantavirus-infected *Sorex* shrews captured at three localities in the Sakha Republic. As noted earlier, hantavirus RNA was found predominantly in male shrews, with evidence of infection in only three female shrews. Pair-wise alignment and comparison of the full-length and partial S-, M- and L-segment sequences from Laxmann’s shrews and flat-skulled shrews showed three genetically distinct hantaviruses. The first was Kenkeme virus (KKMV) strain MSB148794, previously reported from the flat-skulled shrew [14]. The second was Artybash virus (ARTV), harbored by the Laxmann’s shrew [20]. However, based on recent analysis, ARTV should be called SWSV [31]. In addition, the third was a newfound hantavirus, differing by approximately 40% at both the nucleotide and amino acid levels from SWSV and KKMV, and resembling most closely ALTV, previously detected in a Eurasian common shrew from Western Siberia [27].

**Figure 2:**
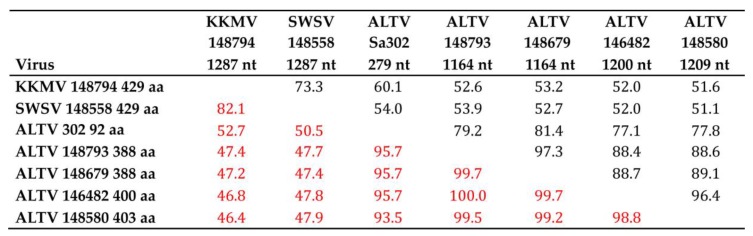
Pairwise alignment and comparison of nucleotide and amino acid S-segment sequences of Kenkeme virus (KKMV Sr422/MSB148794), Seewis virus (SWSV Sca371/MSB148558), Altai virus (ALTV Telet-Sa302) and representative ALTV-like hantaviruses (Sca363/MSB146482, Sca370/MSB148580, Sca402MSB148793, Sr424/MSB148679). Nucleotide sequence similarities are designated in black and amino acid sequence similarities in red.

The full-length 1287-nucleotide S-genomic segment of SWSV and KKMV from the Laxmann’s shrew and flat-skulled shrew, respectively, encoded a 428-amino acid nucleocapsid (N) protein and lacked the additional open reading frame encoding a nonstructural NSs protein, as determined by sequence alignment with cricetid rodent-borne orthohantaviruses. Nearly full-length S-segment sequences of 1164 to 1209 nucleotides, obtained for ALTV-like hantaviruses from three Laxmann’s shrews and one flat-skulled shrew, showed 51.1–53.9% and 46.4–47.9% sequence similarity at the nucleotide and amino acid levels, respectively, with KKMV and SWSV (Figure 2). By contrast, these ALTV-like hantaviruses exhibited amino acid sequence similarity of 98.8–100% (numbers in red) among themselves and approximately 95% similarity with prototype ALTV Telet-Sa302 (Figure 2).

Partial M-segment sequences, amplified from three Laxmann’s shrews (Sca370/MSB148580, Sca377/MSB148458 and Sca402/MSB148793) and a flat-skulled shrew (Sr424/MSB148679), also showed low level nucleotide sequence similarity of 61.3–65.6% with KKMV. By contrast, they exhibited 90.0–91.1% nucleotide sequence similarity and LENV Khekhtsir-Sc67 (which served as a surrogate for prototype ALTV Telet-Sa302 because M segment sequences were unavailable).

**Figure 3:**
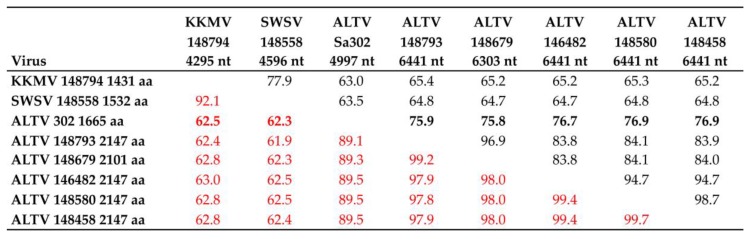
Pairwise alignment and comparison of nucleotide and amino acid L-segment sequences of prototype Kenkeme virus (KKMV Sr422/MSB148794), Seewis virus (SWSV Sca371/MSB148558), Altai virus (ALTV Telet-Sa302) and representative ALTV-like hantaviruses (Sca363/MSB146482, Sca370/MSB148580, Sca377/MSB148458, Sca402/MSB148793 and Sr424/MSB148679).

The nearly full-length L-genomic segments of 6303 to 6441 nucleotides, amplified from four Laxmann’s shrews (Sca363/MSB146482, Sca370/MSB148580, Sca377/MSB148458 and Sca402/MSB148793) and a flat-skulled shrew (Sr424/MSB148679), showed 64.7–65.4% and 61.9–63.0% sequence similarity at the nucleotide and amino acid levels, respectively, with KKMV and SWSV (Figure 3). At the amino acid level (numbers shown in red), these sequences exhibited nearly 90% similarity with prototype ALTV Telet-Sa302 (Figure 3). Thus, there was overall congruence for each genomic segment, suggesting the maintenance and co-circulation of a separate genetic lineage of hantavirus in *Sorex* shrews in the Sakha Republic.

### 3.3. Recombination Analysis

RDP4 Beta 4.36 failed to disclose any consistent evidence of recombination in the S- and L-genomic segments. Although separate regions of potential recombination were found in a few instances, there was no consistency or concordance between the detection methods, calling into question the validity of the identified sequences or the biological significance of recombination versus general heterogeneity in sequence evolutionary rates.

### 3.4. Phylogenetic Analysis

**Figure 4:**
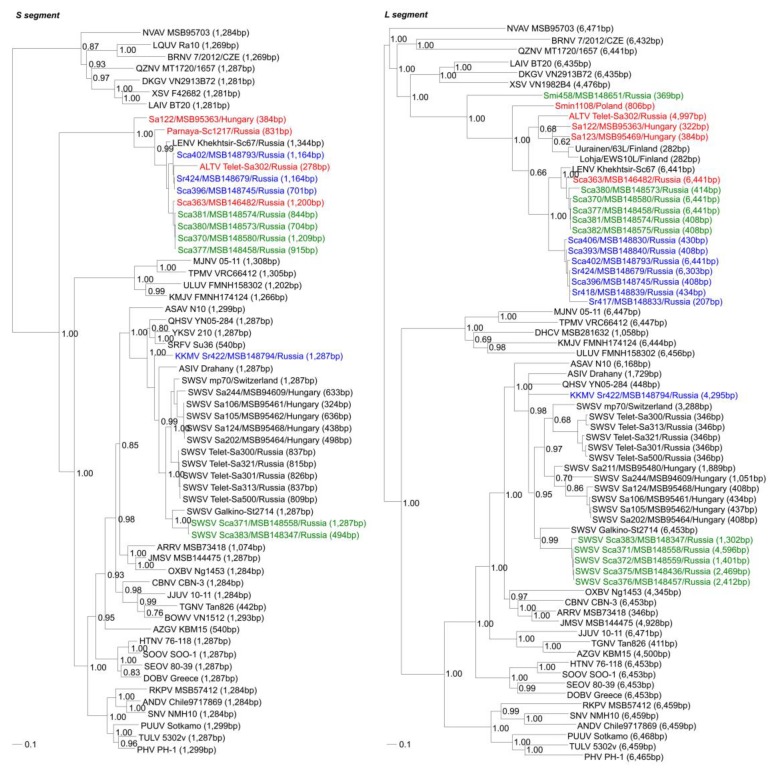
Phylogenetic trees, based on full-length or partial S- and L-segment sequences, generated by the Bayesian Markov chain Monte Carlo estimation method, under the general time reversible model of nucleotide evolution with gamma-distributed rate heterogeneity and invariable sites (GTR+I+Γ), showing geographic-specific clustering of hantaviruses detected in *Sorex caecutiens* and *Sorex roboratus* captured along the Kenkeme River (blue) and Amga River (green). Altai virus (ALTV Telet-Sa302, S: KP657656; L: EU424341) and ALTV-related hantaviruses formed a monophyletic group that shared a common ancestry with mobatviruses, including Láibīn virus (LAIV) strains BT20 (S: KM102247; L: KM102249), Đakrông virus (DKGV) strain VN2913B72 (S: MG663536; L: MG663534), Xuân Sơn virus (XSV) strains F42682, S: KF704709) and VN1982 (L: JX912953), Quezon virus (QZNV) strain MT1720/1657 (S: KU950713; L: KU950715), Brno virus (BRNV) strain 7/2012 (S: KX845678; L: KX845680), and Nova virus (NVAV) strain MSB95703 (S: FJ539168; L: FJ593498), and loanviruses, including Lóngquán virus (LQUV) strain Ra-10 (S: JX465413) and Brno virus (BRNV) strain 7/2012/CZE (S: KX845678; L: KX845680). ALTV and ALTV-related hantaviruses are colored according to the shrew collection sites: Amga River (green); Kenkeme River (blue); taxa shown in red are prototype ALTV Telet-Sa302 from Western Siberia and ALTV-like hantaviruses from Far Eastern Russia (Sca363/MSB146482), Hungary (Sa122/MSB95363 and Sa123/MSB95469) and Poland (Smin1108). Also shown are the phylogenetic positions of prototype Seewis virus (SWSV mp70, S: EF636024; L: EF636026; and SWSV Sca371/MSB148558, S: KM201411; L: KM201413), Kenkeme virus (KKMV Sr422/MSB148794, S: GQ306148; L: GQ306150), as well as other soricine shrew-borne orthohantaviruses, including Ash River virus (ARRV MSB734418, S: EF650086; L: EF619961), Asikkala virus (ASIV Drahany/CZ, S: KC880342; L: KC880348), Azagny virus (AZGV KBM15, S: JF276226; L: JF276228), Bowé virus (BOWV VN1512, S: KC631782), Cao Bằng virus (CBNV CBN-3, S: EF543524; L: EF543525), Jeju virus (JJUV 10-11, S: HQ834695; L: HQ834697), Jemez Springs virus (JMSV MSB144475, S: FJ593499; L: FJ593501), Qiān Hú Shān virus (QHSV YN05-284, S: GU566023; L: GU566021), Sarufutsu virus (SRFV Su36, S: KF700097), Tanganya virus (TGNV Tan826, S: EF050455; L: EF050454) and Yákèshí virus (YKSV 210, S: JX465423), and mole-borne orthohantaviruses, including Asama virus (ASAV N10, S: EU929072; L: EU929078), Oxbow virus (OXBV Ng1453, S: FJ5339166; L: FJ593497), and Rockport virus (RKPV MSB57412, S: HM015223; L: HM015221), and rodent-borne orthohantaviruses, including Andes virus (ANDV Chile9717869, S: AF291702; L: AF291704), Dobrava-Belgrade virus (DOBV/BGDV Greece, S: NC_005233; L: NC_005235), Hantaan virus (HTNV 76-118, S: NC_005218; L: NC_005222), Prospect Hill virus (PHV PH-1, S: Z49098; L: EF646763), Puumala virus (PUUV Sotkamo, S: NC_005224; L: NC_005225), Seoul virus (SEOV HR80-39, S: NC_005236; L: NC_005238), Sin Nombre virus (SNV NMH10, S: NC_005216; L: NC_005217), Soochong virus (SOOV SOO-1, S: AY675349; L: DQ056292), and Tula virus (TULV M5302v, S: NC_005227; L: NC_005226). Also shown are prototype thottimviruses, such as Thottapalayam virus (TPMV VRC66412, S: AY526097; L: EU001330) and Imjin virus (MJNV 05-11, S: EF641804; L: EF641806), as well as presumptive thottimviruses, such as Dahonggou Creek virus (DHCV MSB281632, L: HQ616595), Kilimanjaro virus (KMJV FMNH174124, S: JX193698; L: JX193700), and Uluguru virus (ULUV FMNH158302, S: JX193695; L: JX193697). The recently reported ALTV-like hantavirus sequences of Lena River virus (LENV Khekhtsir-Sc67, S: MH499470; M: MH499471; L: MH499472) are also included. The GenBank accession numbers for the SWSV strains and ALTV-like hantavirus sequences from Hungary and Russia are provided below. The numbers at each node are Bayesian posterior probabilities (>0.70) based on 150,000 trees: two replicate Markov chain Monte Carlo runs, consisting of six chains of 10 million generations each sampled every 100 generations with a burn-in of 25,000 (25%). Scale bars indicate nucleotide substitutions per site.

Phylogenetic trees, based on the coding regions of the full-length and partial S and L segments, and partial M segment, revealed similar topologies using the maximum-likelihood and Bayesian methods. Hantavirus sequences from five Laxmann’s shrews (Sca371/MSB148558, Sca372/MSB148559, Sca375/MSB148436, Sca376/MSB148457 and Sca383/MSB148347) and one flat-skulled shrew (Sr422/MSB148794) segregated into separate clades with SWSV and KKMV, respectively, while the other full-length and partial S-, M- and/or L-genomic sequences from 10 Laxmann’s shrews (Sca363/MSB146482, Sca370/MSB148580, Sca377/MSB148458, Sca380/MSB148573, Sca381/MSB148574, Sca382/MSB148575, Sca393/MSB148840, Sca396/MSB148745, Sca402/MSB148793 and Sca406/MSB148830), three flat-skulled shrews (Sr417/MSB148833, Sr418/MSB148839 and Sr424/MSB148679) and one Eurasian least shrew (Smi458/MSB148651) consistently formed a highly divergent lineage with ALTV Telet-Sa302, which was more closely related to loanviruses and mobatviruses (Figure 4). Specifically, in trees based on the S- and L-segment sequences, *Sorex araneus* from Hungary (Sa122/MSB95363 and Sa123/MSB95469) and Finland (Uurainen/63L and Lohja/EWS10L) and *Sorex caecutiens* (Parnaya-Sc1217, Khekhtsir-Sc67, Sca363/MSB146482, Sca370/MSB148580, Sca377/MSB148458, Sca380/MSB148573, Sca381/MSB148574, Sca396/MSB148745 and Sca402/MSB148793), and *Sorex roboratus* (Sr418/MSB148839 and Sr424/MSB148679) from Russia clustered with prototype ALTV Telet-Sa302 from Western Siberia (Figure 4). Similarly, in the M-segment tree, hantaviruses from three Laxmann’s shrews (Sca370/MSB148580, Sca377/MSB148458 and Sca402/MSB148793) and one flat-skulled shrew (Sr424/MSB148679), as well as a hantavirus from a Eurasian pygmy shrew from Poland (Smin1108), clustered with LENV Khekhtsir-Sc67 (Figure S1). The overall topology of the M and L trees were more similar and suggested that ALTV and ALTV-like hantaviruses belonged to the *Mobatvirus* genus. Geographic-specific clustering was evident for ALTV-related hantaviruses from Laxmann’s shrews and flat-skulled shrews captured along Kenkeme River (blue) and Amga River (green) (Figure 4 and Figure S1).

### 3.5. Host Phylogeny Analysis

The identity of each hantavirus-infected *Sorex* shrew was molecularly confirmed by amplification and sequencing of the full-length 1140-nucleotide cytochrome *b* gene. Phylogenetic trees revealed well-supported lineages according to species.

### 3.6. Virus Isolation

Despite using well-established protocols involving multiple blind passages in Vero E6 cells for 100 days or longer, repeated attempts to isolate these highly divergent lineages of hantaviruses from archival frozen shrew tissues were unsuccessful.

## 4. Discussion

In 2007, we first detected highly divergent hantavirus sequences in Eurasian common shrews captured in Russia and Hungary. A partial L-segment sequence, named ALTV, was deposited in GenBank, but we were unwilling to report this until an expanded database was assembled. In this report, we demonstrate through genetic and phylogenetic analyses of full-length and near full-length sequences of the L- and S-genomic segments, as well as partial M-segment sequences, that genetic variants of ALTV are harbored and are being maintained in Laxmann’s shrews, flat-skulled shrews and Eurasian least shrews in the Sakha Republic in Far Eastern Russia, as well as in Eurasian common shrews in Western Siberia and Hungary. Importantly, these multiple strains of ALTV-like hantavirus are circulating simultaneously with the prototypic host-specific soricine shrew-borne orthohantaviruses. That is, Eurasian common shrews and Laxmann’s shrews harbor SWSV and flat-skulled shrews host KKMV, as well as ALTV-related hantaviruses in the same locality and at the same time.

Apart from the Eurasian common shrew, the Laxmann’s shrew and the flat-skulled shrew, ALTV-related hantavirus sequences were also found in a Eurasian pygmy shrew, captured in Chmiel, in southeastern Poland. The Eurasian pygmy shrew, which is the principal reservoir host of Asikkala virus in Finland [24] and the Czech Republic [48], has also been demonstrated to harbor SWSV in Germany [22], the Czech Republic [22], Finland [24] and Poland [17,26]. This is yet another example of a soricid species serving as the host of more than one hantavirus species. Moreover, the hosting of the same hantavirus species by multiple closely related rodent, soricid, talpid and bat species represents a stark departure from the previously held notion of one-rodent species harboring a single hantavirus species. Thus, the primary reservoir host of ALTV, which has been detected in Eurasian common shrews, Laxmann’s shrews and flat-skulled shrews at high frequency, would be merely speculative. However, the Laxmann’s shrew would be a tentative guess, based on its vast geographic range, extending from Sweden, Finland and Belarus across western Siberia and Mongolia to Far Eastern Russia and China.

Although the gender-specific prevalence of hantavirus infection was not statistically significant among soricine shrews in the Sakha Republic, there was a tendency toward a male predominance. This observation is consistent with the reported overrepresentation of hantavirus infection in male shrews and rodents, including the Ussuri white-toothed shrew [6], Norway rat [49], deer mouse [50,51], and marsh rice rat [52].

Our overall findings confirm those of Ling and colleagues, who previously reported the co-existence of two genetically distinct hantavirus species—SWSV and ALTV-like hantavirus—circulating simultaneously in a single host species, the Eurasian common shrew, in Finland [24]. Their comparative analysis, showing two ALTV-like hantavirus strains (designated Uurainen/63L and Lohja/EWS10L) amplified from Eurasian common shrews, was inferred from the 340-nucleotide L segment of ALTV strain Telet-Sa302 we originally deposited in January 2008 (GenBank EU424341). The phylogenetic analyses of 17 ALTV-like hantavirus variants reported here are now based on the near full-length L segment (4997 nucleotides) of ALTV strain Telet-Sa302. Importantly, the collective data emphasize the widespread geographic distribution and host diversity of ALTV-related hantaviruses, further enriching the complexity of hantavirus evolution and phylogeography [8,9].

The changing global landscape of hantaviruses has prompted a re-examination of previously long-held dogma about their host range, evolutionary origins and phylogeography [8,9]. Based on the rapidly expanding literature of newfound hantaviruses, it is likely that many more hantaviruses will be discovered, possibly in hosts belonging to other taxonomic orders and in unanticipated geographic regions [53]. Moreover, textbook chapters on hantaviruses are being revised and re-written, as more information becomes available about the emergence and pathogenic potential of non-rodent-borne hantaviruses [53,54]. However, despite these advances, some of the persistent uncertainties and conundrums in hantavirus research are the direct consequence of the lack of full-length genomes and the dearth of hantavirus isolates. That is, although referred to as novel viruses, nearly all of the hantaviruses newly identified in shrews, moles and bats exist only as viral sequences.

The isolation of hantaviruses is fraught with difficulty, even from freshly collected tissues. Thus, while disappointing, it is not altogether surprising that we failed to isolate ALTV-like hantaviruses from frozen archival tissues stored since 2006. To date, only three hantaviruses have been isolated from non-rodent hosts: TPMV from the Asian house shrew [1]; MJNV from the Ussuri white-toothed shrew [9]; and Nova virus from the European mole [47]. More innovative approaches are urgently needed to isolate hantaviruses, including the establishment of cell lines from tissues of reservoir hosts, the engineering of cells with specific virus-entry receptors, and the development of three-dimensional organoid cultures. Until such time that ALTV, ALTV-like hantaviruses and other non-rodent-borne hantaviruses are isolated and propagated in culture, their biology and pathogenic potential will remain speculative at best. Thus, the road ahead is laden with challenges, but also endless opportunities and unlimited possibilities. Above all, strong partnerships between healthcare providers, public health workers, veterinarians, ecologists, museum curators and pathologists will be vital for the identification and rapid diagnosis of previously unrecognized infectious diseases, caused by newfound hantaviruses.

## 5. Conclusions

Eurasian common shrews, Laxmann’s shrews and flat-skulled shrews, captured at the same time and in the same location in Hungary and Russia, were each found to harbor hantaviruses belonging to two separate and highly divergent lineages. Pair-wise alignment and phylogenetic analysis of partial and full-length S-, M- and/or L-segment sequences indicated the co-existence and maintenance of two distinct hantavirus lineages in these closely related syntopic soricine shrew species. These findings suggest possible ancient host-switching events from another reservoir. Although ALTV was originally detected in the Eurasian common shrew and ALTV-like hantaviruses have been found in Eurasian common shrews from Finland and Hungary, the primary reservoir host of ALTV is unknown. However, since the vast geographic distribution of ALTV-like hantaviruses coincides with the geographic range of the Laxmann’s shrew, this shrew species is the likely candidate. Alternatively, ALTV might represent an ancestral hantavirus lineage that may have subsequently diversified within the Soricini tribe in Eurasia, based on being detected in *Sorex araneus*, *Sorex caecutiens*, *Sorex minutissimus*, *Sorex minutus* and *Sorex roboratus*. Co-circulation of hantaviruses in the same host species also raises the distinct possibility of co-infection and reassortment as a mechanism for rapid evolutionary change [25,55,56,57,58,59]. Our analysis did not show evidence of recombination, but the possibility of reassortment would exist if shrews were infected concurrently with KKMV and ALTV or SWSV and ALTV. Finally, our data did not allow definitive classification of ALTV and ALTV-like hantaviruses into one of the existing four genera of the subfamily *Mammantavirinae*. Whether this means there might be a fifth genus warrants further intensive investigation.

## Figures and Tables

**Table 1:** Prevalence of hantavirus RNA and occurrence of divergent hantavirus sequence in *Sorex* shrews from Eurasia.

Country	Collection Site	Species	Year	No. Tested	HantavirusRNA Positive	Divergent Sequence
Finland	Etelä-Suomen Lääni	*Sorex araneus*	1982	10	4	0
	Lappi	*Sorex araneus*	1982	3	1	0
	Oulun Lääni	*Sorex araneus*	1982	9	7	0
Hungary	Györ-Sopron-Moson	*Sorex araneus*	1997	19	11	1
	Nógrád	*Sorex araneus*	1997	3	1	1
	Zala	*Sorex araneus*	2000	44	3	0
Poland	Chmiel	*Sorex araneus*	2010	11	4	0
		*Sorex minutus*	2010	7	1	1
	Huta Dłutowska	*Sorex araneus*	2011	9	2	0
	Kurowice	*Sorex araneus*	2013	13	5	0
Russia	Teletskoye Lake	*Sorex araneus*	2007	9	6	1
	Irkutsk City	*Sorex daphaenodon*	2007	2	2	0
	**Amga River**	***Sorex caecutiens***	**2006**	**19**	**10**	**5**
		***Sorex minutissimus***	**2006**	**5**	**1**	**1**
	**Kenkeme River**	***Sorex caecutiens***	**2006**	**24**	**4**	**4**
		***Sorex daphaenodon***	**2006**	**4**	**0**	**0**
		***Sorex roboratus***	**2006**	**12**	**4**	**3**
	**Lena River**	***Sorex caecutiens***	**2006**	**6**	**1**	**1**
		***Sorex tundrensis***	**2006**	**5**	**0**	**0**

Samples for the present study are shown in **bold** type.

**Table 2:** Summary of hantavirus sequences in *Sorex* shrews captured in the Sakha Republic in 2006.

Site	Species	Virus	MSB	Sex	Date	Hantavirus Sequence		
						S	M	L
Amga	*Sorex caecutiens*	SWSV	148347	male	14 August 2006	956	731	751, 1304, 1046
River	*Sorex caecutiens*	SWSV	148436	male	12 August 2006		1094	2414, 476
	*Sorex caecutiens*	SWSV	148457	male	12 August 2006		1095	2414, 476
	*Sorex caecutiens*	SWSV	148558	male	10 August 2006	1627	1088	4598
	*Sorex caecutiens*	SWSV	148559	female	10 August 2006		971	2414, 476
	*Sorex caecutiens*	ALTV	148458	male	12 August 2006	1385	558	6535
	*Sorex caecutiens*	ALTV	148573	male	14 August 2006	704		400, 409
	*Sorex caecutiens*	ALTV	148574	male	14 August 2006	804		409
	*Sorex caecutiens*	ALTV	148575	male	14 August 2006			409
	*Sorex caecutiens*	ALTV	148580	male	09 August 2006	1600	568	6535
	*Sorex minutissimus*	ALTV	148651	male	14 August 2006			362
Kenkeme	*Sorex caecutiens*	ALTV	148745	male	20 August 2006	703		241, 409
River	*Sorex caecutiens*	ALTV	148793	female	20 August 2006	1600	890	6533
	*Sorex caecutiens*	ALTV	148830	male	21 August 2006			409
	*Sorex caecutiens*	ALTV	148840	male	20 August 2006	686		409
	*Sorex roboratus*	ALTV	148679	male	21 August 2006	1664	907	6533
	*Sorex roboratus*	ALTV	148833	male	20 August 2006			208
	*Sorex roboratus*	ALTV	148839	female	20 August 2006			409
	*Sorex roboratus*	KKMV	148794	male	20 August 2006	1640	1002	4304
Lena River	*Sorex caecutiens*	ALTV	146482	male	02 August 2006	1600		6500

Hantavirus designation in third column: ALTV, Altai virus; KKMV, Kenkeme virus; SWSV, Seewis virus. MSB is the Museum of Southwestern Biology mammal catalog number. Originally, based on the high sequence similarity with Artybash virus (ARTV), we had classified the hantaviruses in several *Sorex caecutiens* as ARTV. However, recent analysis has shown that ARTV is a genetic variant of SWSV [31]. Accordingly, SWSV is used in Table 2 and Figure 2, Figure 3 and Figure 4.

## Supplementary Materials

The following are available online at https://www.mdpi.com/1999-4915/11/9/857/s1. Table S1: Oligonucleotide primers for amplification of SWSV, ARTV, KKMV and ALTV. Figure S1: Phylogenetic tree, based on M-segment sequences of ALTV-like hantaviruses.

## Nucleotide Sequences

GenBank accession numbers for the ALTV-like hantavirus sequences include Sa122/MSB95363 (S: GQ293127; L: GQ293104) and Sa123/MSB95469 (L: GQ293105) from Hungary; and Sca363/MSB146482 (S: KM361043; L: KM361055), Sca370/MSB148580 (S: KM361044; M: KM361051; L: KM361056), Sca377 MSB148458 (S: KM361045; M: KM361052; L: KM361057), Sca380/MSB148573 (S: KM361046; L: KM361058), Sca381/MSB148574 (S: KM361047; L: KM361059), Sca382/MSB148575 (L: MN401400), Sca393/MSB148840 (L: MN401399), Sca396/MSB148745 (S: KM361050; L: KM361060), Sca402/MSB148793 (S: KM361048; M: KM361053; L: KM361061), Sca406/MSB148830 (L:MN401398), Sr417/MSB148833 (L: MN401401), Sr418/MSB148839 (L: KM361062), Sr424/MSB148679 (S: KM361049; M: KM361054; L: KM361063) and Parnaya-Sc1217 (S: KM362174) from Russia, as well as ALTV-like hantavirus strains Uurainen/63L (L: KJ136623) and Lohja/EWS10L (L: KJ136638) from Finland. Also, new orthohantavirus sequences include Seewis virus (SWSV) SWSV Sa105/MSB95462 (S: GQ293124; L: GQ293097), SWSV Sa106/MSB95461 (S: GU186442; L: GQ293098), SWSV Sa124/MSB95468 (S: GQ293128; L: GQ293106), SWSV Sa202/MSB95464 (S: GQ293137; L: GQ293117), SWSV Sa211/MSB95480 (L: GQ293121), SWSV Sa244/MSB94609 (S: CQ293138; L: CQ293123) from Hungary, and SWSV Sca371/MSB148558 (S: KM201411; L: KM201413), SWSV Sca372/MSB148559 (L: KM201414), SWSV Sca375/MSB148436 (L: KM201416), SWSV Sca376/MSB148457 (L: KM201418), SWSV Sca383/MSB148347 (S: KM201419; L: KM201421) from the Sakha Republic, and SWSV Telet-Sa300 (S: GQ284578; L: EU424334), SWSV Telet-Sa301 (S: GQ284577; L: EU424335), SWSV Telet-Sa313 (S: GQ284579; L: EU424336), SWSV Telet-Sa321 (S: GQ284575; L: EU424337), SWSV Telet-Sa500 (S: GQ284576; L: EU424338) from Teletskoye Lake in the Altai Republic.
