# Supplementary material for: Highly Divergent Genetic Variants of Soricid-Borne Altai Virus (Hantaviridae) in Eurasia Suggest Ancient Host-Switching Events
Source: Viruses. 2019 Sep 14;11(9):857. doi: 10.3390/v11090857 (PMC6783933; doi:10.3390/v11090857)
Supplement: Supplementary file 1 [file viruses-11-00857-s001.pdf]

Table S1. Oligonucleotide primers used to amplify the S, M and L segments of soricine shrew-borne hantaviruses.

Segment	Primer Name	Sequence	Length
SML	OSM55(F)	TAG TAG TAG ACT CC	14
S	PHS-3endR	TAG TAG TAT ACT CCT TGA AAA GC	23
	HTN-S6	AGC TCN GGA TCC ATN TCA TC	20
	Cro2R	ANG AYT GRT ARA ANG ANG AYT TYT T	25
	Cro2F	AGY CCN GTN ATG RGW GTN RTY GG	23
	CBS-3'endR	TAG TAG TAK RCT CCY TRA A	19
	SO-S970R	GGY SCA CHN GCA AAN ACC CA	20
	SO-S620R	CCA GGW GTA ATC TCW TCA GCC	21
	SO-S450F	TAC ATG TTN ACA ACT AGA GGG AG	23
	SO-S430F	CAA TNG TCT TGA AGG CNT TKT ACA T	25
	SO-S1040F	TGC AGG ATA TNA GGA ACC TGA	21
	Hanta-S587R	CCW GGT GTN ANY TCD TC	17
	HTN-S1F	TAG TAG TAG ACT YCN TRM DDA	21
	Han-S604F	GCH GAD GAR HTN ACA CCN GG	20
	Han-S974R	TCN GGN GCH CHN GCA AAN AHC CA	23
	Han-S3R	TAG TAG TAN NCT CCY TRW ACA	21
	Han-S1260R	CDG GRT CCA TRT CAT CHC CNA	21
	Han-S694F	CCN GTN ATG RGN GTN ATH GGN TT	23
	Han-S952F	TGG DTN TTT GCN DRD GSN CC	20
M	TM-2957R	GAA CCC CAD GCC CCNTCY AT	20
	OSV697F	GGA CCA GGT GCA DCT TGT GAA GC	23
	T-M1485R	CCA GCC AAA RCA RAA TGT	18
	HTN-M-2353F	TAC WGG YTG YAC TGC RTG TGG	21
	HTN-M1370F	ACH AAA ACH YTA GTN ATW GG	20
	HTN-M1470F	GGN TTY CAT GGN TGG GCN AC	20
	HTN-M1970F	TNT GGG CWG CHA GTG CNG A	19
	HTN-M2020R	CCA TGD GCA KTR TCA NTC CA	20
	HTN-M1520F	TTG GNT GGN TNY TAA THC C	19
	Han-M2631R	CAT NAY RTC NCC RGG RTC NCC	21
	Han-M2957R	GAR CCC CAN GCN CCN TCW AT	20
L	PHL-173F	GAT WAA GCA TGA YTG GTC TGA	21
	PHL-2818R	GGW CCA TAW GAA ATG TAC TCT TC	23
	PHL-2111F	CAG TCW ACA RTT GGT GCA AGT GG	23
	PHL-3endR	TAG TAG TAG TAT GCA CCG GAA	21
	HAN-L-F1	ATG TAY GTB AGT GCW GAT GC	20
	HAN-L-R1	AAC CAD TCW gTY CCR TCA TC	20
	HAN-L-F2	TGC WGA TGC HAC NAA RTG GTC	21
	HAN-L-R2	GCR TCR TCW GAR TGR TGD GCA A	22
	RPLV-L-366F	RGT CAC TGT GAC AGY WGA TGT	21
	RPLV-L-4837R	CYG TMC CYT CWA CAT TAC CTT G	22
	RPLV-L-577R	MCC GKC ATT HCG YCT ACT WGG C	22
	TMUS-L-3R	TAG TAG TAT GCT CCG RNR AA	20
	SL-1458R	AKT ANA TGS CCT ATA TGC CAT GC	23
	363L-1050R	AAC TCA CTC AAC ATG TCT CTG	21
	SO-L4680F	GAY ATA TCA ATA CCA GAR GTY ATG AG	26
	SO-L4637F	GAT TCW AGG ACA TTR AAR GAG	21
	SO-L2540R	CCT GAC ATA CCC TTY ART GA	20
	SO-L2290R	TTN ARN GCC CAT TCN ACA GTC TC	23
	SO-L1730R	TAA ATC AAT RCT CAT TAC	18
	SO-L1060F	ATG AAA TTA GGY AAT GCT GA	20
	SO-L340R	TAG TTG TCT GGK GTC ATC TT	20
	Han-L3R	TAG TAG TAK GCT CCG NRR	18
	Han-L3000R	GCN GAR TTR TCN CCN GGN GAC CA	23
	Han-L2935R	GTN GCR TCN GCA CTN ACA TAC AT	23
	Han-L2707F	CAR MGN ACN GAR GCN GAT RGN GG	23
	Han-L1900F	ATG AAR NTN TGT GCN ATN TTT GA	23

Abbreviations: A, Adenine; B, C or G or T; C, Cytosine; D, A or G or T; G, Guanine; H, A or C or T; I, Inosine; K, G or T; M, A or C; N, any nucleotide; R, A or G; S, G or C; T, Thymine; V, A or C or G; W, A or T; Y, C or T.

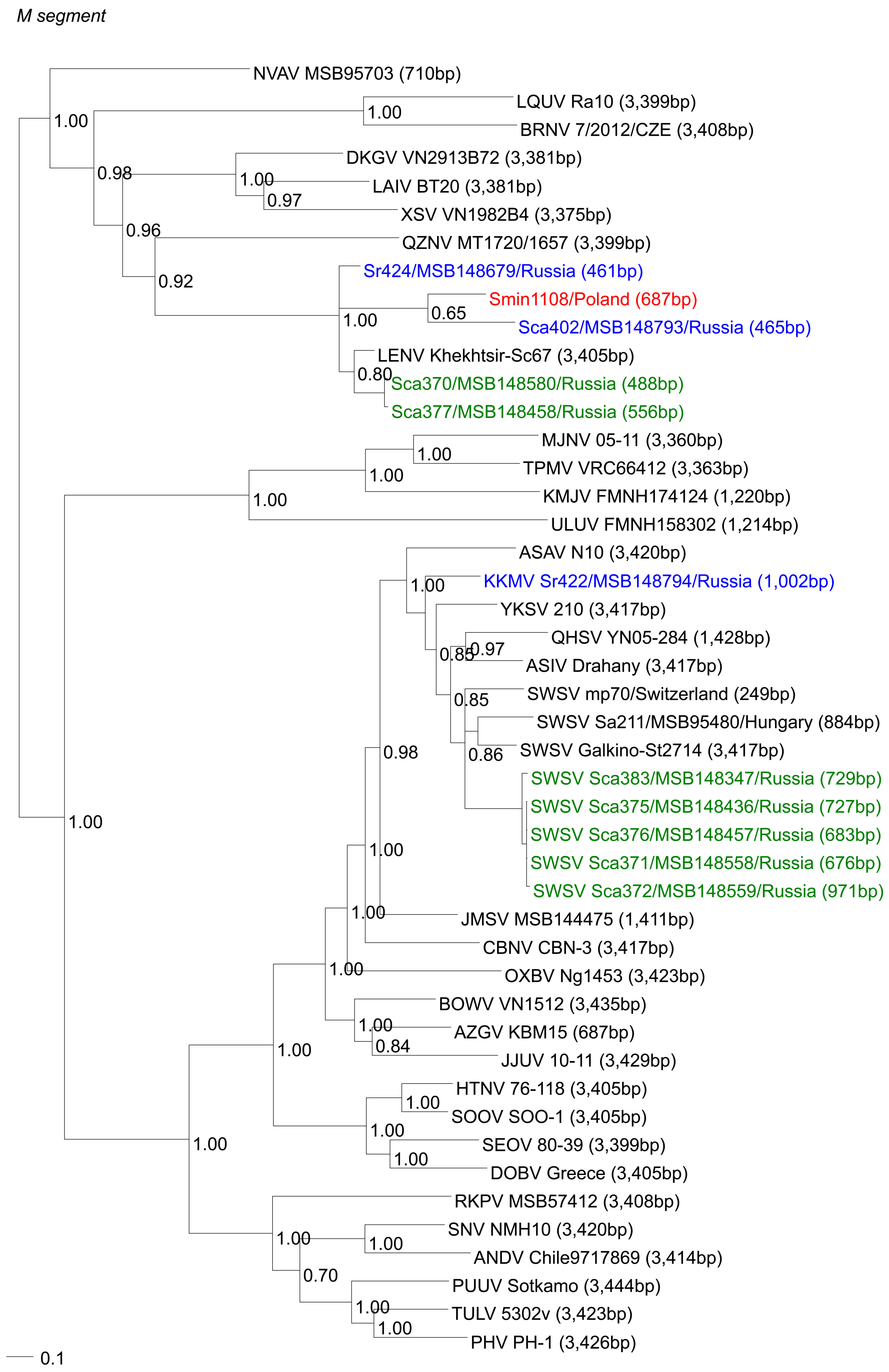

Figure S1. Phylogenetic tree, based on M-segment sequences, generated by the Bayesian Markov chain Monte Carlo estimation method, under the GTR+I+Γ model of evolution, showing geographic-specific clustering of hantaviruses from *Sorex caecutiens* and *Sorex roboratus* captured along the Kenkeme River (blue) and Amga River (green). Altai virus (ALTV)-related hantaviruses formed a monophyletic group that shared a common ancestry with loanviruses, such Lóngquán virus (LQUV Ra-10: JX465396) and Brno virus (BRNV 7/2012/CZE:

KX845679), and mobatviruses, such as Xuân Sơn virus (XSV VN1982B4: KU976427), Quezon virus (QZNV MT1720: KU950714), Đakrông virus (DKGV VN2913B72: MG663535), Láibīn virus (LBV BT20: KM102248) and Nova virus (NVAV MSB95703: HQ840957). The phylogenetic positions of ALTV-related hantaviruses are shown in relationship to prototype Seewis virus (SWSV mp70: EF636025), genetic variants of SWSV (formerly classified as Artybash virus, namely, SWSV Galkino-St2714: MG913806; SWSV Sca371/MSB148558: KM201412; SWSV Sca383/MSB148347: KM201420; SWSV Sca375/MSB148436: KM201415; SWSV Sca376/MSB148457: KM201417), Kenkeme virus (KKMV Sr422/MSB148794: GQ306149), as well as other soricine shrew-borne orthohantaviruses, including Asikkala virus (ASIV Drahany/CZ: KC880345), Azagny virus (AZGV KBM15: JF276227), Bowé virus (BOWV VN1512: KC631783), Cao Bằng virus (CBNV CBN-3: EF543526), Jeju virus (JJUV 10-11: HQ834696), Jemez Springs virus (JMSV MSB144475: FJ593500), Qiān Hú Shān virus (QHSV YN05-284: GU566022), and Yákèshí virus (YKSV 210: JX465403), and mole-borne orthohantaviruses, including Asama virus (ASAV N10: EU929073), Oxbow virus (OXBV Ng1453: FJ539167), and Rockport virus (RKPV MSB57412: HM015519), and rodent-borne orthohantaviruses, including Andes virus (ANDV Chile9717869: AF291703), Dobrava-Belgrade virus (DOBV/BGDV Greece: NC_005234), Hantaan virus (HTNV 76-118: NC_005219), Prospect Hill virus (PHV PH-1: X55129), Puumala virus (PUUV Sotkamo: NC_005223), Seoul virus (SEOV HR80-39: NC_005237), Sin Nombre virus (SNV NMH10: NC_005215), Soochong virus (SOOV SOO-1: AY675353), and Tula virus (TULV M5302v: NC_005228). Also shown are prototype thottimviruses, such as Thottapalayam virus (TPMV VRC66412: EU001329) and Imjin virus (MJNV 05-11: EF641805), as well as presumptive thottimviruses, such as Kilimanjaro virus (KMJV FMNH174124: JX193699), and Uluguru virus (ULUV FMNH158302: JX193696). The recently reported ALTV-like hantavirus sequence of Lena River virus (LENV Khekhtsir-Sc67: MH499471) is also included. The GenBank accession numbers for the ALTV-like hantavirus sequences are provided following the Conclusions section. The numbers at each node are Bayesian posterior probabilities (>0.70) based on 150,000 trees: two replicate Markov chain Monte Carlo runs, consisting of six chains of 10 million generations each sampled every 100 generations with a burn-in of 25,000 (25%). Scale bars indicate nucleotide substitutions per site.
